# English Learning Stress, Self-Efficacy, and Burnout among Undergraduate Students: The Moderating Effect of Mindfulness and Gender

**DOI:** 10.3390/ijerph192315819

**Published:** 2022-11-28

**Authors:** Liling Xu, Huahua Wang, Jiaxin Chen, Yiwen Zhang, Zhiqi Huang, Chengfu Yu

**Affiliations:** 1 School of Foreign Studies, South China Normal University, Guangzhou 510631, China; 2 Department of Psychology, Research Center of Adolescent Psychology and Behavior, School of Education, Guangzhou University, Guangzhou 510006, China

**Keywords:** English learning stress, English learning self-efficacy, English learning burnout, mindfulness, undergraduate students

## Abstract

Research has indicated that English learning stress contributes significantly to English learning burnout among undergraduate students. However, knowledge of the mediating and moderating mechanisms underlying this relationship is limited. To bridge this gap, a moderated mediation model was constructed to examine whether English learning self-efficacy mediated the relationship between English learning stress and English learning burnout. Furthermore, this study analyzed whether the mediated relationship was moderated by mindfulness and gender. A total of 1130 Chinese undergraduate students (mean age = 20.84 years, SD = 1.57 years) reported their experiences regarding English learning stress, English learning self-efficacy, English learning burnout, and mindfulness. After controlling for covariates, the results revealed that English learning self-efficacy mediated the positive link between English learning stress and English learning burnout among both men and women. Moreover, the findings demonstrated that the indirect link was moderated by mindfulness among male undergraduate students. However, the moderating effect of mindfulness was not significant among the women in this study. The implications of these findings for future research, and the development of intervention and prevention of English learning burnout are discussed.

## 1. Introduction

Learning burnout refers to feelings of exhaustion experienced by students during the learning process. Learning burnout is accompanied by cynical and detached attitudes toward studies and school activities, as well as a low sense of achievement in learning [1]. English learning burnout refers to feelings of exhaustion and a lack of interest in learning English, which can induce a range of detrimental outcomes. For instance, English learning burnout can harm the physical and mental health of students and may lead to poor learning performance and dropout [2,3,4]. English learning burnout could be more serious in Chinese students based on the natural language environment and test-oriented education in China [5]. English learning is compulsory for Chinese undergraduates and is evaluated using nationwide standardized tests (i.e., College English Test Band 4/6). Furthermore, these scores are used as a graduation or recruitment criterion [6]. Thus, such a unique context necessitates the exploration of English learning burnout and its potential mechanism among Chinese undergraduate students.

As one of the largest English learning groups, Chinese students experience immense academic stress regarding English learning [7]. Thus, this study focused on the impact of English learning stress on English learning burnout among Chinese undergraduates. According to the transactional model of stress and coping [8], the long-term effects of stress can develop into chronic stress symptoms and cause a burnout response. Furthermore, learning stress can reduce academic failure tolerance resulting in academic burnout [9]. Heavy learning stress could also damage the willingness of students to engage in exploratory learning activities, subsequently increasing the risk of learning burnout. Moreover, empirical studies support the view that undergraduate students who experience more learning stress face a higher risk of learning burnout [9,10,11]. For example, Wang et al. [10] investigated 1119 Chinese undergraduate students from eight different universities and found that learning stress had a significant positive predictive effect on learning burnout. Researchers have begun investigating the link between learning stress and learning burnout among undergraduate students. However, the mediating and moderating mechanisms underlying this relationship remain largely unexplored. To bridge the gap, this study investigated the association between English learning stress and English learning burnout based on English learning self-efficacy, mindfulness, and gender among Chinese undergraduate students.

### 1.1. The Mediating Effect of English Learning Self-Efficacy

Learning self-efficacy refers to an individual’s belief in his or her ability to succeed in scholastic activities [12]. In addition, learning self-efficacy is considered the main factor for determining the effectiveness of an individual’s second language learning and ability to use it [13]. According to the transactional model of stress and coping [8], stressors can influence an individual’s cognitive appraisals and coping styles and exacerbate negative outcomes. Therefore, cognitive appraisals (e.g., learning self-efficacy) comprise a key explanatory mechanism in the link between English learning stress and negative outcomes (e.g., English learning burnout). To date, research has not directly examined the mediating role of learning self-efficacy in the link between English learning stress and undergraduate students’ English learning burnout. However, several studies provide indirect evidence that supports this mediation process.

Undergraduate students with heavier perceived English learning stress could suffer from impaired learning self-efficacy. Chronic learning stress often leads to chronic negative effects such as anxiety, depression, and hopelessness [14]. Furthermore, excessive academic tasks as well as frequent academic failures and inadequacies can also damage undergraduate students’ learning motivation [15]. Bandura [12] emphasized that aversive arousal states that are associated with stress could be interpreted as a sign of potential failure and consequently decrease the individual’s self-efficacy perceptions. Consistent with these views, empirical studies have reported that learning stress could jeopardize undergraduate students’ learning self-efficacy [16,17]. For example, Ling and Lim [17] found that learning stress was negatively associated with learning self-efficacy in a sample of 610 Chinese university students.

English learning burnout can also occur among undergraduate students with low English learning self-efficacy. For example, individuals with a low level of English learning self-efficacy are more likely to adopt avoidance strategies [18], exacerbating their risk of English learning burnout. Furthermore, they are less likely to be resilient to learning failures and adversity, consequently exhibiting higher levels of learning burnout [19]. Moreover, empirical studies have found that learning self-efficacy has a significant predictive effect on the persistence of learning burnout among undergraduate students [10,20,21]. Previous studies have also emphasized the mediating effect of learning self-efficacy in the relationship between learning stress and learning burnout among adolescents [18,22]. However, this mediating effect has not been explored among undergraduate students. Thus, this study explored whether English learning self-efficacy mediates the relationship between English learning stress and undergraduate students’ English learning burnout.

### 1.2. Mindfulness and Gender as Moderators

Although English learning stress is generally considered a risk factor for English learning burnout among undergraduate students, not all students are influenced equally by English learning stress. Therefore, it is important to examine variables that may moderate the link between English learning stress and English learning burnout. Thus, this study examined whether the indirect pathway would vary as a function of undergraduate students’ mindfulness. Mindfulness describes a state where an individual consciously pays attention to his or her experience, thoughts, and feelings of the present moment without judgment [23]. As a positive personality factor, mindfulness can result in desirable achievements when learning a second or foreign language [24]. For example, researchers have found that individuals with high levels of mindfulness exhibit more English learning motivation [25], less English learning anxiety [26], and less academic burnout [27,28].

According to the mindfulness reperceiving model [29], mindfulness can promote a fundamental psychological perspective shift called “reperceiving”, which is a meta-mechanism of action that yields change and positive outcomes. Through the process of mindfulness reperceiving, individuals can dis-identify from the content of consciousness and obtain a more clear and objective view of moments and experiences. Consequently, these individuals participate in less reflexive behavior [29]. Therefore, mindfulness could moderate the relationship between English learning stress, English learning self-efficacy, and English learning burnout. Empirical studies have supported this opinion. For instance, Park et al. [30] found that mindfulness moderated the relationship between stress (including academic stress) and depression among university students. Specifically, university students with higher mindfulness were less likely to be affected by stress. Likewise, Yoo [31] found that mindfulness moderated the relationship between self-efficacy and job burnout among hotel employees. The relationship between self-efficacy and job burnout was stronger in hotel employees with lower levels of mindfulness, whereas the relationship between self-efficacy and job burnout was weaker in those with higher levels of mindfulness. To date, no previous research has examined whether mindfulness is a protective factor that buffers the adverse impact of English learning stress on English learning burnout among undergraduate students.

The moderating effect of mindfulness could also be gender specific. Existing research reveals that gender influences the favorable effects of mindfulness [32,33]. Meanwhile, studies have found that male students demonstrate higher mindfulness and more learning burnout than female students [34,35]. Luo et al. [35] found that gender moderated the relationship between learning adjustment and learning burnout among university students. In contrast to the female students, higher learning maladjustment was more strongly related to higher learning burnout in male university students. However, there is limited knowledge of gender differences in the context of English learning stress and English learning burnout among undergraduate students. This presents an important research question that can contribute to designing appropriate interventions to alleviate English learning burnout in undergraduate students. Therefore, the present study explored whether the beneficial contribution of mindfulness within the context of English learning stress and undergraduate students’ English learning burnout differs according to gender.

### 1.3. The Present Study

A moderated mediation model was used to examine three main hypotheses addressing how English learning stress works and whether all undergraduate students are vulnerable to English learning stress.

**Hypothesis** **1:** *English learning self-efficacy would mediate the relationship between English learning stress and English learning burnout among undergraduate students*.

**Hypothesis** **2a:** *Mindfulness would moderate the indirect pathways from English learning stress to English learning burnout among undergraduate students*.

**Hypothesis** **2b:** *The hypothesized associations would differ by gender*.

## 2. Materials and Methods

### 2.1. Participants

This study sample comprised 1130 undergraduate students (406 men and 724 women) recruited from two universities in southern China. All the participants were non-English majors. The ages of the participants ranged from 17.83 to 24.83 years (Mean age = 20.84 years, SD = 1.57 years). The participants completed a survey designed to collect information regarding demographic characteristics, English learning stress, English learning self-efficacy, English learning burnout, and mindfulness.

### 2.2. Measures

#### 2.2.1. English Learning Stress

English learning stress was assessed with the Chinese version of the English Learning Stress Questionnaire [36]. The measure contains four items. Participants were asked to report the extent to which they experienced stress related to English learning in the past year (e.g., I feel pressured by the English course examination). The scores were rated on a five-point scale ranging from 1 to 5 (1 = never and 5 = always). An average score for all items was calculated, with higher scores indicating higher levels of English learning stress. The questionnaire was used in a previous study with Chinese undergraduate students and demonstrated good reliability and validity [36]. The Cronbach’s α of the scale in this study was 0.91.

#### 2.2.2. English Learning Self-Efficacy

English learning self-efficacy was assessed with the Chinese version of the English Learning Self-Efficacy Questionnaire [37]. The measure contains four items. Participants were asked to report their self-efficacy in English learning over the past year (e.g., I think I have the ability to solve the difficulties I have encountered in English learning). Responses were scored on a five-point scale ranging from 1 to 5 (1 = do not agree at all and 5 = perfectly agree). An average score for all items was calculated, with higher scores indicating higher English learning self-efficacy. The Cronbach’s α of the scale in this study was 0.78.

#### 2.2.3. English Learning Burnout

English learning burnout was assessed with the Chinese version of the English Learning Burnout Questionnaire [38]. The measure contains thirteen items. Participants were asked to report how burned out they felt about learning English in the past year (e.g., I have a hard time staying enthusiastic about my studies). Responses were scored on a five-point scale ranging from 1 to 5 (1 = do not agree at all and 5 = completely agree). An average score for all items was calculated, with higher scores indicating a higher level of burnout. The questionnaire has been used in a previous study with Chinese undergraduate students and demonstrated good reliability and validity [38]. The Cronbach’s α of the scale in this study was 0.76.

#### 2.2.4. Mindfulness

Mindfulness was measured with the Chinese version of the Mindfulness Questionnaire [39]. The measure contains four items. Participants were asked to rate the frequency of events in the past year (e.g., I find myself doing things without paying attention). Responses were scored on a six-point scale ranging from 1 to 6 (1 = almost never and 6 = almost always). An average score for all items was calculated, with higher scores indicating higher levels of mindfulness. The Cronbach’s α of the scale in this study was 0.93.

### 2.3. Control Variables

Considering the age of undergraduate students, English learning proficiency and neuroticism have a significant effect on their learning burnout [40,41,42]. Consequently, these variables were included as control variables. English examination scores were used as a measure of English learning proficiency; higher scores indicated greater English learning proficiency. Neuroticism was measured with the Neuroticism subscale of the Chinese Big Five Personality Inventory (CBF-PI-15) [43], which contains three items (e.g., I often worry that something bad is going to happen). Responses to items utilized a six-point scale ranging from 1 to 6 (1 = do not agree at all and 6 = strongly agree). An average score was calculated for all items, with higher scores indicating higher levels of neuroticism. The Cronbach’s α of the scale in this study was 0.91. All Chinese students use the same English textbooks in secondary education and all non-English majors take the same English courses in university education. Thus, we did not control the students’ background in English in secondary education and the requirements of English courses in university education.

### 2.4. Research Procedures and Statistical Analysis

Informed consent was obtained from each participant before completing the questionnaires online, which required approximately 20 min. The SPSS 25.0 was used for data analysis. First, descriptive analyses were conducted to examine associations between the study variables and covariates. Second, the SPSS PROCESS macro (Model 4) [44] was used to test the mediation model proposed in this study. Subsequently, the SPSS PROCESS macro (Model 73) [44] was used to test the moderation model proposed in this study. Finally, when the moderating effect was significant, a simple slope plot was created based on the values obtained by adding or subtracting the moderating variable by one standard deviation.

## 3. Results

### 3.1. Preliminary Analyses

Descriptive statistics and correlations of all variables are presented in Table 1. As expected, English learning stress was positively associated with English learning burnout. In contrast, English learning stress was negatively associated with English learning self-efficacy and mindfulness. Furthermore, English learning self-efficacy was positively associated with mindfulness, whereas English learning burnout was negatively associated with English learning self-efficacy and mindfulness.

### 3.2. Testing for Mediation

Model 4 of the PROCESS macro [44] was used to test the mediating effect of English learning self-efficacy on the relationship between English learning stress and English learning burnout (Hypothesis 1). The results (see Model 1 in Table 2) indicated that English learning stress negatively predicted English learning self-efficacy (*β* = −0.40, *p* < 0.001) after controlling for covariates. Furthermore, the results (see Model 2 in Table 2) indicated that English learning self-efficacy negatively predicted English learning burnout (*β* = −0.28, *p* < 0.001). Moreover, English learning stress directly predicted English learning burnout (*β* = 0.13, *p* < 0.001) after controlling for the covariates (see Model 2 in Table 2). The bootstrapping analyses indicated that the indirect effect of English learning stress on English learning burnout through English learning self-efficacy was significant (*β* = 0.11, 95% CI [0.08, 0.15]). Thus, these findings suggest that undergraduate students who experienced greater English learning stress were prone to having lower levels of English learning self-efficacy, exacerbating their risk of English learning burnout. The mediated effect accounted for 46.22% of the total effects. Thus, the first hypothesis was supported: English learning self-efficacy mediated the relationship between English learning stress and English learning burnout.

### 3.3. Testing for Moderated Mediation

**Figure 1:**
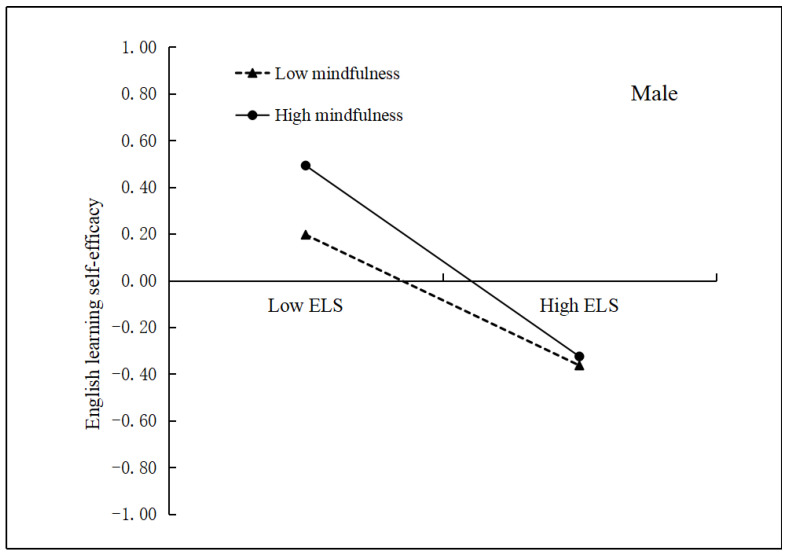
English learning self-efficacy as a function of English learning stress and mindfulness among male undergraduate students. ELS = English learning stress.

**Figure 2:**
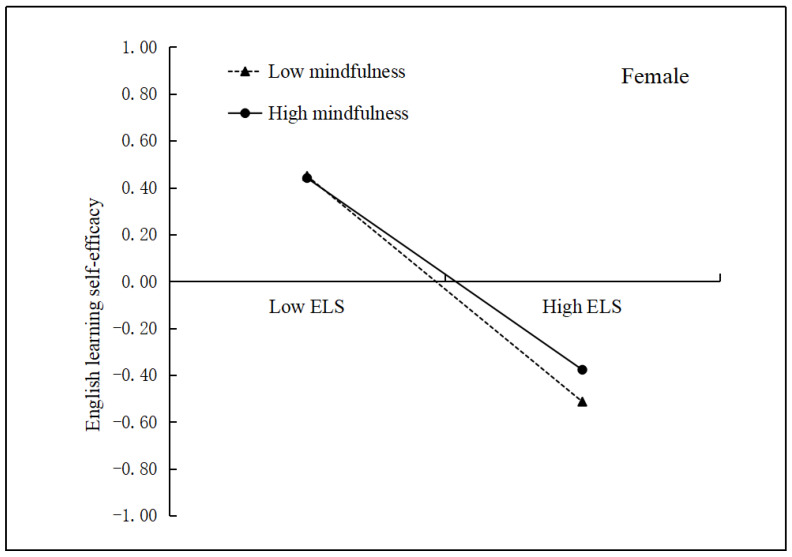
English learning self-efficacy as a function of English learning stress and mindfulness among female undergraduate students. ELS = English learning stress.

Model 73 of the PROCESS macro [44] was used to test the moderating effects of mindfulness and gender on the mediation model. As presented in Table 3, Model 1 indicates that English learning stress negatively predicted English learning self-efficacy (*β* = −0.44, *p* < 0.01) after controlling for covariates. Furthermore, both mindfulness (*β* = 0.03, *p* > 0.05) and gender (*β* = 0.07, *p* > 0.05) positively predicted English learning self-efficacy. However, mindfulness did not have a significant moderating role in the first part of the mediation process (i.e., English learning stress → English learning self-efficacy; *β* = 0.04, *p* > 0.05). Moreover, a two-way interaction between gender and English learning stress (*β* = 0.07, *p* > 0.05) or mindfulness (*β* = 0.06, *p* > 0.05) was not observed. However, a significant three-way interaction between English learning stress, mindfulness, and gender was found to predict English learning self-efficacy (*β* = −0.10, *p* < 0.05). To further investigate the interaction, the relationship between English learning stress and English learning self-efficacy was plotted across low and high levels of mindfulness for men and women (Figure 1 and Figure 2). Consequently, simple slope tests showed that the relationship between English learning stress and English learning self-efficacy was moderated by mindfulness among the male participants (*β* = −0.06, *SE* = 0.03, *p* < 0.05; Figure 1). Figure 1 exhibits that the negative relationship between English learning stress and English learning burnout was significant for men with low mindfulness (*β* = −0.28, *SE* = 0.05, *p* < 0.01). In contrast, this association was much stronger for men with high mindfulness (*β* = −0.42, *SE* = 0.06, *p* < 0.01). Conversely, no significant moderating effect of mindfulness was found for women (*β* = 0.04, *SE* = 0.03, *p* > 0.05; Figure 2). These findings indicate that mindfulness moderated the first part of the mediation process (i.e., English learning stress → English learning self-efficacy) among the men in this study.

**Figure 3:**
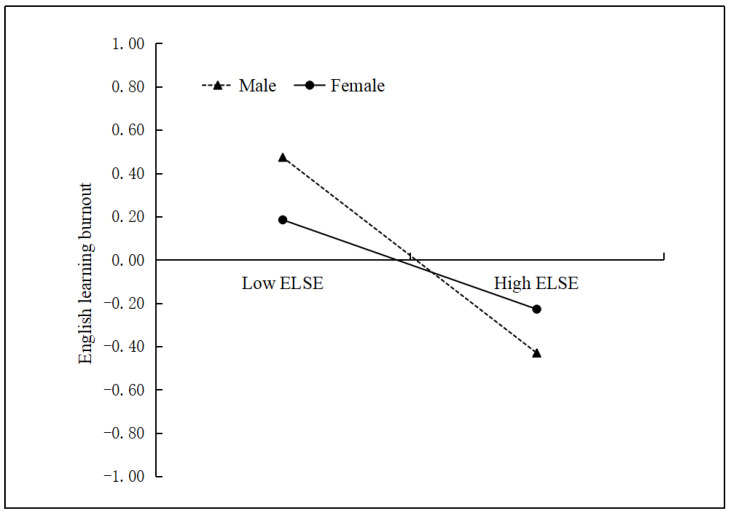
English learning burnout as a function of English learning self-efficacy and gender. ELSE = English learning self-efficacy.

Model 2 in Table 3 indicates that English learning self-efficacy negatively predicted English learning burnout (*β* = −0.31, *p* < 0.01). Furthermore, after controlling for covariates and English learning self-efficacy, English learning stress did not directly predict English learning burnout (*β* = 0.05, *p* > 0.05). Moreover, mindfulness negatively predicted English learning burnout (*β* = −0.30, *p* < 0.01). However, gender was not associated with English learning burnout (*β* = −0.05, *p* > 0.05). Furthermore, there was no significant moderating role of mindfulness in the second part of the mediation process (i.e., English learning self-efficacy → English learning burnout) or any residual direct relationship (i.e., English learning stress → English learning burnout) (all *p* > 0.05), and there was no significant three-way interaction (*p* > 0.05). However, a significant moderating role of gender was found in the second part of the mediation process (*β* = 0.11, *p* < 0.05). To further investigate the interaction, simple slope tests were conducted. Figure 3 depicts that English learning self-efficacy negatively predicted English learning burnout, and this relationship was much stronger in women (*β* = −0.33, *SE* = 0.04, *p* < 0.01) than in men (*β* = −0.21, *SE* = 0.05, *p* < 0.01). Finally, the bias-corrected percentile bootstrap results indicated that the mediating effect of English learning self-efficacy was stronger for women with low mindfulness (conditional indirect effect = 0.17, *SE* = 0.02, 95% CI [0.12, 0.22]) than for men (conditional indirect effect = 0.05, *SE* = 0.02, 95% CI [0.01, 0.10]). Moreover, self-efficacy was stronger for women with high mindfulness (conditional indirect effect = 0.14, *SE* = 0.02, 95% CI [0.10, 0.18]) than for the men in this study (conditional indirect effect = 0.07, *SE* = 0.03, 95% CI [0.02, 0.13]).

## 4. Discussion

### 4.1. The Mediating Effect of English Learning Self-Efficacy

In support of Hypothesis 1, the results showed that English learning self-efficacy mediated the relationship between English learning stress and English learning burnout. Therefore, English learning self-efficacy should be considered as a critical carrier linking English learning stress and English learning burnout among Chinese undergraduate students. This finding supports the transactional model of stress and coping [8], because English learning stress may threaten English learning self-efficacy leading to negative outcomes such as English learning burnout. Impaired English learning self-efficacy is not only the outcome of English learning stress, but also a catalyst for English learning burnout among Chinese undergraduate students. The current study is the first to demonstrate how the transactional model of stress and coping helps explain the relationship between English learning stress and English learning burnout.

The results are also consistent with previous studies [18,22]. Undergraduate students with higher English learning stress had a lower level of English learning adaptability, and they were more likely to experience frustration, despair, and hopelessness [14]. These feelings may contribute to decreased self-efficacy in learning English, leading students to believe that they cannot complete learning tasks [12]. When students deem that they cannot efficiently use their energy, they develop negative expectations regarding good learning results [18]. Thus, they may experience English learning burnout. However, lower English learning stress may help undergraduate students develop higher self-confidence and self-efficacy in English learning [17,45]. Feeling efficacious can not only promote undergraduate students to develop more favorable outcome expectations, but also motivate them to act and make progress toward academic goals and subsequent accomplishments [45]. Thus, they would be less likely to experience English learning burnout.

### 4.2. The Moderating Role of Mindfulness and Gender

The results revealed that there was a significant three-way interaction between English learning stress, mindfulness, and gender in predicting English learning self-efficacy, which partially supported Hypothesis 2. Specifically, the moderating effect of mindfulness on the relationship between English learning stress and English learning self-efficacy was significant for male but not female undergraduates. For men, consistent with the mindfulness reperceiving model [29], undergraduate students with high levels of mindfulness showed higher English learning self-efficacy than those with low levels; however, this difference was larger when they reported a low level of English learning stress. This result is consistent with the findings of Han and Xu [46], who found that learning self-efficacy improved in men who had participated in a mindfulness-based cognitive therapy intervention. However, in the present study, the relationship did not differ in women based on whether they were high or low in mindfulness. Possible explanations regarding this finding are as follows. Male undergraduates are weaker in foreign language learning, and thus experience more difficulty and feel more incompetent in English learning than females [47]. Therefore, men may experience greater English learning stress and mindfulness may attenuate the stress effectively. Notably, this result innovatively validated the mindfulness reperceiving model [29], indicating that mindfulness can interact with English learning stress, protect learning self-efficacy, and effectively alleviate learning burnout. According to current knowledge, this is the first study to provide sufficient evidence confirming that interaction between English learning stress and mindfulness affects English learning burnout among Chinese undergraduate students.

However, this study found that mindfulness did not moderate the relationship between English learning self-efficacy and English learning burnout for both genders. One possible explanation may be that learning self-efficacy plays an irreplaceable role in the academic accomplishments of individuals [12]; thus, low learning self-efficacy may be a contributing factor in the experience of burnout for both genders regardless of their levels of mindfulness. Interestingly, this study also found that gender significantly moderated the path of learning self-efficacy to learning burnout. Specifically, the negative correlation between English learning self-efficacy and English learning burnout was stronger for women than for men. Possible explanations for this result are as follows. Compared with men, women are more likely to evaluate and judge their own English learning ability [48]. Consequently, women become more vulnerable to the internal cognition (e.g., self-efficacy) [49]. Therefore, when female undergraduates perceive that they are incompetent in English learning, they are more likely to develop English learning burnout.

### 4.3. Limitations and Future Directions

There are several limitations to consider in the current study. First, the cross-sectional design of this study did not underscore the causal relationships between English learning stress, English learning self-efficacy, and English learning burnout. Thus, future studies should consider using a longitudinal tracking design from the perspective of development to clarify this causal relationship. Second, the participants of this study represent a small portion of Chinese undergraduate students, which is not very representative. Thus, the generalizability of the current study results ought to be considered. Consequently, future research should consider selecting more diverse samples for replication research. Third, the data in this study were collected in the form of self-reports from undergraduate students, which might have yielded recall biases. Future research should consider multi-method and multi-informant approaches to further examine the robustness of our findings. Finally, gender in this study was a strict binary variable that excluded transgender people. To address this limitation, future research should expand the definition of gender outside of the strict binary presentation to produce more interesting and applicable findings.

### 4.4. Implications of This Study

Despite the limitations of this study, the findings provide a scientific basis and guidance for interventions and prevention of English learning burnout among undergraduate students. First, increased stress associated with learning English comprises an important risk factor for English learning burnout among Chinese undergraduate students. Therefore, emphasis should be placed on reducing the learning stress and burden experienced by undergraduate students, as well as creating a free and open English learning environment for them. Second, the current findings can help practitioners understand the relationship between English learning self-efficacy and English learning burnout among undergraduate students. Particularly, this finding reveals that actively paying attention to and improving students’ learning self-efficacy could alleviate learning burnout among undergraduate students. Timely and positive feedback is considered a very effective tool. Third, enhanced mindfulness among undergraduate students can mitigate the risk of learning burnout. Specifically, training and daily practice represented by mindfulness-based cognitive therapy and dialectical behavior therapy can significantly improve the level of individual mindfulness in the prevention and intervention of experiencing burnout [50]. Finally, this study found very important gender differences associated with English learning burnout among undergraduate students. As mentioned above, women are better at foreign language learning than men. Thus, it may be helpful to generally improve English teaching in primary and secondary school prior to tertiary education, and encourage and reinforce boys' foreign language learning skills.

## 5. Conclusions

In conclusion, this study constructed and tested a moderated mediation model on the relationship between English learning stress and English learning burnout among Chinese undergraduate students. Furthermore, the study provided an in-depth investigation of the underlying psychological mechanism. These findings underscore the importance of considering English learning self-efficacy, mindfulness, and gender to improve our understanding of how and when English learning stress impacts English learning burnout among undergraduate students.

## Figures and Tables

**Table 1:** Means, standard deviations, and correlation coefficients for all variables.

Variables	1	2	3	4	5	6	7	8
1. Age	–							
2. Gender	0.00	–						
3. Neuroticism	0.01	0.02	–					
4. EP	0.03	−0.12 **	0.03	–				
5. ELS	0.01	0.06 *	0.26 **	−0.29 **	–			
6. ELSE	0.03	0.02	−0.21 **	0.47 **	−0.53 **	–		
7. Mindfulness	0.01	0.04	−0.55 **	0.05	−0.25 **	0.20 **	–	
8. ELB	−0.07 *	0.01	0.40 **	−0.26 **	0.38 **	−0.45 **	−0.46 **	–
*Mean*	20.27	–	3.44	3.30	3.00	2.93	4.10	2.95
*SD*	1.48	–	1.20	1.50	1.03	0.79	1.15	0.44

Note: Gender was dummy coded 1 = male, 0 = female. EP = English proficiency; ELS = English learning stress; ELSE = English learning self-efficacy; ELB = English learning burnout. * *p* < 0.05, ** *p* < 0.01.

**Table 2:** Testing for the mediation effect of English learning self-efficacy.

Variables	Model 1 (ELSE)				Model 2 (ELB)			
	*β*	*SE*	*t*	95% CI	*β*	*SE*	*t*	95% CI
Age	0.02	0.02	1.23	[−0.01, 0.05]	−0.04	0.02	−2.49 *	[−0.07, −0.01]
Gender	0.04	0.02	1.81	[−0.01, 0.09]	−0.01	0.02	−0.38	[−0.06, 0.04]
Neuroticism	−0.10	0.02	−4.07 **	[−0.14, −0.05]	0.31	0.03	12.07 ***	[0.26, 0.36]
EP	0.35	0.02	14.23 ***	[0.30, 0.39]	−0.09	0.03	−3.17 **	[−0.14, −0.03]
ELS	−0.40	0.03	−16.11 ***	[−0.45, −0.35]	0.13	0.03	4.44 ***	[0.07, 0.19]
ELSE					−0.28	0.03	−8.78 ***	[−0.34, −0.21]
*R* ^2^	0.40				0.33			
*F*	184.89 ***				109.20 ***			

Note: Gender was dummy coded 1 = male, 0 = female. EP = English proficiency; ELS = English learning stress; ELSE = English learning self-efficacy; ELB = English learning burnout. * *p* < 0.05, ** *p* < 0.01, *** *p* < 0.001.

**Table 3:** Testing for moderated mediation.

Variables	Model 1 (ELSE)				Model 2 (ELB)			
	*β*	*SE*	*t*	95% CI	*β*	*SE*	*t*	95% CI
Age	0.02	0.02	1.34	[−0.01, 0.05]	−0.04	0.02	−2.55 *	[−0.07, −0.01]
Neuroticism	−0.07	0.03	−2.48 *	[−0.12, −0.01]	0.16	0.03	5.58 **	[0.10, 0.21]
EP	0.35	0.02	14.39 ***	[0.30, 0.39]	−0.09	0.03	−3.38 **	[−0.14, −0.04]
ELS	−0.44	0.03	−13.32 **	[−0.50, −0.37]	0.05	0.04	1.30	[−0.03, 0.12]
Mindfulness (MO)	0.03	0.03	0.81	[−0.04, 0.09]	−0.30	0.03	−8.83 **	[−0.37, −0.24]
Gender (G)	0.07	0.05	1.31	[−0.03, 0.17]	−0.05	0.05	−1.00	[−0.15, 0.05]
ELS×MO	0.04	0.03	1.23	[−0.02, 0.09]	−0.07	0.04	−1.99	[−0.14, −0.00]
ELS×G	0.07	0.05	1.49	[−0.02, 0.17]	0.14	0.06	2.38 *	[0.02, 0.25]
MO×G	0.06	0.05	1.21	[−0.04, 0.15]	0.03	0.05	0.61	[−0.07, 0.13]
ELS×MO×G	−0.10	0.04	−2.44 *	[−0.18, −0.02]	0.04	0.05	0.91	[−0.05, 0.14]
ELSE					−0.31	0.04	−8.05 **	[−0.38, −0.23]
ELSE×MO					−0.05	0.04	−1.41	[−0.12, 0.02]
ELSE×G					0.11	0.06	1.97 *	[0.00, 0.22]
ELSE×MO×G					0.04	0.05	0.96	[−0.05, 0.14]
*R^2^*	0.40				0.39			
*F*	76.04 **				51.51 **			

Note: Gender was dummy coded 1 = male, 0 = female. EP = English proficiency; ELS = English learning stress; ELSE = English learning self-efficacy; ELB = English learning burnout. * *p* < 0.05, ** *p* < 0.01, *** *p* < 0.001.

## Data Availability

The data presented in this study are available on request from the corresponding authors (C.Y.).
